# Unlocking the Power of Artificial Intelligence and Big Data in Medicine

**DOI:** 10.2196/16607

**Published:** 2019-11-08

**Authors:** Christian Lovis

**Affiliations:** 1 Division of Medical Information Sciences University Hospitals of Geneva Geneva Switzerland; 2 Department of Radiology and Medical Informatics University of Geneva Geneva Switzerland

**Keywords:** medical informatics, artificial intelligence, big data

## Abstract

Data-driven science and its corollaries in machine learning and the wider field of artificial intelligence have the potential to drive important changes in medicine. However, medicine is not a science like any other: It is deeply and tightly bound with a large and wide network of legal, ethical, regulatory, economical, and societal dependencies. As a consequence, the scientific and technological progresses in handling information and its further processing and cross-linking for decision support and predictive systems must be accompanied by parallel changes in the global environment, with numerous stakeholders, including citizen and society. What can be seen at the first glance as a barrier and a mechanism slowing down the progression of data science must, however, be considered an important asset. Only global adoption can transform the potential of big data and artificial intelligence into an effective breakthroughs in handling health and medicine. This requires science and society, scientists and citizens, to progress together.

## Introduction

Most of the daily news and recently published scientific papers on research, innovations, and applications in artificial intelligence (AI) refer to what is known as machine learning—algorithms using massive amounts of data and various methodologies to find patterns, support decisions, make predictions, or, for the deep learning part, self-identify important features in data. However, AI is a complex concept to grasp, and most people have little understanding of what it really is. AI was founded as an academic discipline in 1956 and, despite its youth, already has a rich history [1,2]. In more than 60 years of exploration and progress, AI has become a large field of research and development involving multidisciplinary approaches to address many challenges, from theoretical frameworks, methods, and tools to real implementations, risk analysis, and impact measures. The definition of AI is a moving target and changes over time with the evolution of the field. Since its early days, the field of AI has allowed the development of many techniques supporting decision support and prediction, as it is usually made by humans. As early as 1958, a perceptron was expected to be able “to walk, talk, see, write, reproduce itself and be conscious of its existence,“ which led a large scientific controversy between neural network and symbolic reasoning approaches [3]. The landscape of AI research includes knowledge representation and engineering; rule-based and symbolic reasoning; temporal reasoning and planning; sensing and perception; learning; evolutionary, emerging, social behaviors; and the ability to move and manipulate objects, to name the most important [4]—deep machine learning with autonomous features extraction. It is the point of view taken in the paper, acknowledging however that, more recently, there is a trend to restrict AI to the latter, autonomous deep machine learning. As a consequence of the wide landscape, the field draws at large through philosophy, mathematics, information sciences, computer science, psychology, anthropology, social sciences, linguistics, and many others. For some experts and visionary people such as Ray Kurzweil, deep machine learning will allow building of an artificial general intelligence that is able to develop itself autonomously and to have the capacity to understand or learn any intellectual task that a human being can, and even go far beyond the limits of human intelligence [5], but most experts would agree that there are some big missing pieces and it is still a long way off, despite recent potential important advances in quantic computing [6]. A recent white paper published by the European Commission and authored by the members of the High-Level Expert Group on AI provides, in a few pages, a good overview on what AI is, its main capabilities, applicable expectations, and disciplines involved [7].

Taking into the field of AI at large, it is important to emphasize that AI is already broadly used today in medicine. Decision support based on knowledge engineering and rule-based systems are implemented widely in computerized provider order entry (CPOE) worldwide. Advanced signal processing is implemented in pacemakers or defibrillators to take decisions, in cochlear-implants with man-machine interfaces, in electrocardiograms to provide signal analysis and automated diagnosis, etc.

The AI field in itself is aspirational and is expected to contribute significantly to medicine, from research to citizen-centered health. Machine learning and deep learning has led most recent major breakthroughs in AI, such as sound (speech and music) recognition and image (face, radiology, pathology, dermatology, etc) recognition, and in gaming. Recently, image recognition has almost reached a level of maturity through which it can be used and developed by nonexperts in AI [8,9]. However, the hype around AI in these last few years has built high expectations and similarly high fears. There are still very few systems based on autonomous deep learning that have emerged widely in the commercial market.

The world of AI could roughly be summarized in three sequential and superposed acts:

Act 1: Humans teach machines to handle data and information.

Act 2: Humans teach expertise to machines.

Act 3: Humans teach machines to learn alone.

## Challenges

There are many challenges that need to be addressed in the field of AI, when it comes to medicine. Most of them are not exclusive to medicine and health, but their addition makes the goals significantly much harder to reach.

### Bayesian Trap

Medicine and health determinants, in general, are characterized by their usually fundamental Bayesian property. In the Bayesian probability approach, a prior probability is required to evaluate the strength of the prediction.

Most of what is used in medicine, notably but not exclusively, to establish diagnosis, falls in the Bayesian approach. For example, if a person has a fever, what is the probability of it being the flu? If a person has a high measurement of blood sugar, what is the probability of it being diabetes? To illustrate the Bayesian trap, let us take a simple example—a pregnancy test. This is a simple test; it can be positive or negative. Let us imagine that we use a classical test, which has 99% sensitivity and 95% specificity. If 100 tests are performed for 100 persons and 5 turn out to be positive, the question is, know how many of them turn out to be pregnant women. This question is defined as determining the positive predictive value of a positive test, and it gives the probability of a positive test to be really signing the presence of the factor the test is testing. To answer this question, we need to know the prior probability of being pregnant in the tested population. To understand this, imagine that 100% men were tested; in this case, none of the 5 positive tests would correspond to a pregnant woman. Similarly, if all persons tested are pregnant woman, then all 5 positive tests would correspond to pregnant women. If the prior probability is around 1%, then applying the Bayesian rules returns that the probability to be pregnant when a test is reported positive is about 17%. This means that about 4 of 5 tests are false positive. At the other end, if the prior probability is around 20% (ie, a woman with several factors suggesting a potential pregnancy), the probability of a positive test to be a true positive is above 80%. Thus, less than one test out of five is a false-positive. The example shows the major consequences of the prior probability in Bayesian situations.

These are the consequences of AI. The models must take into account prior probability in the population they are used. This should be better understood even when reporting results in the literature, often limited to specificity and sensitivity. Another consequence only becomes visible when several focused and near-to-perfect systems are used together in complex cases. For example, having many systems, each with its own false-positive rate can end up with consolidated systems that have the sum of all false-positives. This has been shown well with decision support system in CPOE, with a very high rate of false-positive alerts, especially with patients receiving complex drug therapies [10,11].

### Regulatory Labyrinth

Most diagnostic or therapeutic means used nowadays in medicine have to go through complex regulatory frameworks to get market approval. The regulatory agencies mostly base their decisions on safety, evidence, and added value. In addition, medicoeconomic assessments are often used by health agencies according to various dimensions such as quality-adjusted life year and burden of the disease, by using indicators such as disability-adjusted life-years [12-14]. These decisions thus have economical and legal consequences, including accountability. The role of regulatory agencies is discussed, especially around topics that are getting into the market, such as in image recognition [15]. For example, a call for inputs for “Artificial Intelligence and Machine Learning in Software as a Medical Device“ has been launched by the Federal Drug Administration [16]. This is an important aspect, as regulatory agency support is an important asset in building trust for most care professionals to use medical tools and for companies to invest in robust products ready for the market. However, this requires us to define a clear regulatory framework, appropriate evaluation processes, and benchmark tools without blocking innovation [17].

### Education and Practice Gap

Medicine is a science with numerous tools and devices, from stethoscopes to scalpels, microscope to scanners, scores, guidelines, etc. Most of these tools and devices require education and sometimes very specific certification processes for care professionals that use them, not to speak about a good experience. This should be also the case for software, algorithms, and other decision support systems. However, this is not the case. Education to use software and understand systems as important as the computerized patient records is often minimal. When it is about big data and AI, education on the topic is worse, usually inexistent. There are only very few medical schools that teach the use of AI to future health professionals. AI should become mandatory teaching in all medical schools in the world as a priority. Experts have been raising the question since 20 years, but it has received real focus only recently [18-22]. In 5-10 years, when current young students will be starting their clinical activities, machine learning based on data science will have become embedded in many activities, devices, and software and its use, misuse, and overuse and consequences on patients and accountability will depend on how users will master it [23].

### Data Quality Chiasm

Data quality is a recurring topic of discussion when it comes to big data and analytics. One of the characteristics of the big data era is that data are often used for a purpose that differs from the one that motivated data acquisition. This is a notable difference with traditional hypothetic-deductive scientific approaches in medicine, where a hypothesis leads to a methodology design, which itself will lead to specific data acquisition. In the big data era, the primary goal of data-producing processes is often completely independent from possible use of the data. It is interesting to emphasize that long-term clinical cohorts and long-term biobanks face similar challenges. Designing long-term cohorts and building metadata framework and standard operating procedures for biobanking are important challenges, as they have to project usages that will be made years after the initial design.

These questions have led to a consequent literature addressing the question of data quality and secondary usage of clinical data. However, most of this work tries to describe dimensions able to assess the “intrinsic” or “absolute” quality of data [24-28]. Another approach could be to adopt a “fit-for-purpose” approach, which considers only the quantitative and descriptive properties of data, allowing further processing. The “qualitative” properties of any dataset can only be assessed in conjunction with a specific secondary usage. This means that the same dataset will be appropriate to answer some scientific questions and not appropriate to answer others. The data are not “good” or “bad” by themselves; they are “good” or “bad” when used in a specific context: the “fit-for-purpose” assessment. This is one of the major objectives of the FAIR data initiative, which aims at insuring “*a posteriori*” data usability (see below).

An unexpected consequence of the “data quality chiasm” is its influence on modifying acquisition processes, especially in clinical contexts. One often hears sentences such as “the quality of clinical data is not good enough for research.” As a result, there is a constant pressure to move toward more structured data acquisition processes. For example, the RECIST (Response Evaluation Criteria In Solid Tumors) guidelines are meant to standardize the radiologic evaluation criteria in solid tumors oncological trial treatments. This has been successfully developed for trials. Use of RECIST requires good experience to avoid interobserver variability, which can be as high as 20% [29-31]. This assessment has been adapted to reflect changes in radiological response, for example, in immunotherapies where the size of tumors can increase despite good therapeutic response [32]. Unfortunately, there is growing pressure to extend the use of RECIST and other similarly structured staging guidelines beyond clinical trials for all radiological staging to improve the capacity to use standard clinical care for therapeutic assessment. As a consequence, this leads to a high time pressure on operational activities of radiology departments and an increasing number of inexperienced people using these types of staging. With the progression of natural interfaces such as voice recognition and natural language processing and their increased daily use in a growing number devices, I would argue in favor of avoiding artificial structuring many data acquisition processes and keep the data in their most natural form, exploiting more natural interactions such as voice and text and developing strong natural language processing tools that can be applied to produce structured information in a postprocessing step. This will allow reprocessing of all narratives whenever needed by new structured resources required.

## Quest for Truth

Many aspects of the landscape of artificial intelligence require a good idea of what is true. Knowledge engineering builds the graph of the “known” or the “relevant” such as it is made in SNOMED CT (Systematized Nomenclature of Medicine - Clinical Terms) or the Open Biological and Biomedical Ontology Foundry [33,34]. The same applies with rule-based techniques or symbolic reasoning, which need to be able to express rules, that is, truth in a formalized way, but also in supervised machine learning approaches, which require having training sets that express truth, at least a probabilistic truth. There are a lot of expectations in these approaches, especially when combing them [35,36], but all of them, except unsupervised deep machine learning, require some sources of truth, which leads to the fundamental question of finding the sources of truth in life sciences and the level of evidence supporting that truth. At the first glance, it seems to be a trivial question. However, the “truth” is often “lost in text” because for most of it, the sources rely on complex narratives that contextualize the messages they convey. In addition, the “truth” is very diluted. For example, with more than 2500 papers indexed daily in Medline/PubMed [37], it is nearly impossible for an expert to catch everything published in its own research field. Finally, and by nature, science is evolving, and thus, scientific “truth” of what was true once may no more be true today. For example, it was clear until recently that there are two types of lymphocytes—the B and T cells. However, a recent paper from Rizwan et al [38] describes a new type of lymphocyte, bearing characteristics of both B and T cells, which may play a role in driving autoimmunity in some diseases such as diabetes [38]. Sources of truth and their characterizations, such as the level of evidence or their context of use, are increasingly important. This should be available to all, similar to Cochrane [39], covering all area of life sciences; maintained; and in machine-readable form.

## Building Trust

In science, trust is strongly related to building evidence. Trust is important in not only the scientific community, but also at large, to build adoption, political support, and public acceptance. In summer 2019, a survey published by the Pew Research Center showed a positive trend among the public: science acts for the good, but with concerns about integrity, transparency, and bias. Overall, 86% of Americans say they have at least “a fair amount” of confidence in scientists [40]. One of the challenges is that scientific reliability has often been confused with trustworthiness [41]. Scientific evidence can be very strong, such as for immunization or Web-based health information, but the trust can be much lower [42,43]. There are many dimensions that have been discussed in building trust in science, but they can be summarized in three concepts, one for the scientists and the organizations, one for the objects of the research, and one for the processes. Integrity is first and most important and covers scientific integrity, funding, conflict of interests, etc. Transparency must be present for the motivation, outcomes, and process. Finally, methodologies applied to handle the processes must be strong and robust. Building evidence requires many dimensions to be taken into account, such as bias, generalizability, reproducibility, and explainability. Some challenges are more difficult in big data and AI. Proper control of data acquisition and flow is usually more difficult than that in traditional controlled studies. The consequence is that the data have specific properties, which are not always well managed, such as selection biases. Sometimes, the assumptions constraining the use of analytical tools are not well understood, such as homoscedasticity for many statistical tests. In addition, deep machine learning is facing the challenges of precise reproducibility and explainability. The latter is currently the object of numerous works, trying to understand intermediate representation of data in neural networks that can predict and explain their behavior. Explainability and interpretability are often used interchangeably. Interpretability is the extent to which it is possible to predict how the system will behave, given a change in input or algorithmic parameters. On the other hand, explainability is the extent to which the internal mechanics of the deep learning system can be understood and thus explained. Molnar [44] published a very good overview of the problem in an open book available on GitHub. However, explainability might not be the best road to raise global trust in deep machine learning approaches, especially when the explanations themselves are hard to explain. Some other dimensions such as transparency, reproducibility, or uncertainty qualifications might be more effective [45]. For example, in *Science* in 2018, Hutson [46] reported a survey of 400 artificial intelligence papers presented at major conferences, with only 6% including code for the algorithms and 30% test data, thus considerably limiting reproducibility possibilities [46].

## FAIR Data Hope

The FAIR Guiding Principles are guidelines to make data discoverable and processable by both humans and machines. They were first published by Wilkinson et al [47]. The FAIR Guiding Principles are based on a set of criteria listed in Textbox 1:

**FAIR data criteria.**

**Findable**

- (Meta)data are assigned a globally unique and persistent identifier
- Data are described with rich metadata (defined below)
- Metadata clearly and explicitly include the identifier of the data described
- (Meta)data are registered or indexed in a searchable resource

**Accessible**

- (Meta)data are retrievable by their identifier using a standardized communications protocol
- The protocol is open, free, and universally implementable
- The protocol allows for an authentication and authorization procedure, where necessary
- Metadata are accessible, even when the data are no longer available

**Interoperable**

- (Meta)data use a formal, accessible, shared, and broadly applicable language for knowledge representation
- (Meta)data use vocabularies that follow FAIR Principles
- (Meta)data include qualified references to other (meta)data

**Reusable**

Meta(data) are richly described with a plurality of accurate and relevant attributes:

- (Meta)data are released with a clear and accessible data usage license
- (Meta)data are associated with detailed provenance
- (Meta)data meet domain-relevant community standards

Several frameworks have been defined to assess and evaluate the compliance to FAIR criteria, such as the FAIR maturity tools [48,49]. As such, FAIR data do not imply that data are in the Open Data space [50]. Access can be restricted, such as in the Harvard Dataverse, and this is an important point to emphasize. There might be a lot of restriction to have data or metadata available in the Open Data space, because of national regulation, privacy protection, intellectual property, etc. FAIR data do not make data available; they make data usable under the condition that it is authorized.

The FAIR initiative is crucial. It illustrates the movement of data from objects to assets initiated this last decade and described in the essay of Sabina Leonelli [51] recently published in *Nature*.

The initiative promotes the use of rich metadata framework, compliant to standards and formal descriptions. It promotes the use of free and open resources for descriptive information and protocols. It allows us to build a framework of shareable resources that can be used for processing, without actually sharing the data in an open space. FAIR allows building of a framework that is inclusive with all data sources, including those that are subject to authorization, with clear and open protocols.

## Privacy – New Deal

In the era of big data, privacy requires special attention. Usual paradigms of limiting access to deidentified information are becoming less effective to protect privacy. Increasing heterogeneous data sources and richness of data about each of us, associated with data linkage techniques, strongly increases the possibility of reidentification, including anonymized data [52-57]. The challenge and potential impacts are even bigger for genetic information [58-60]. There is no good technical solution that can harmonize the challenge of preserving privacy and answering the increasing need of data-driven science for accessing large genomic et phenotypic datasets, and there are many ongoing ethical and legal discussions [61-66]. Interestingly, this is not restricted to science, and the same applies to patients’ needs for health information [67]. There is a need for better global education about implications and risks of privacy, citizen, policy makers, students, research community, and all stakeholders. A recent scoping review [68] has shown that the understanding of anonymization and de-deidentification is heterogeneous in the scientific community [68]. Discrimination is one of the major risks in privacy breaches, and disclosing privacy information can have many consequences [69-71], including in reimbursement and insurance coverage [72,73]. It is important to find the right path between naïve positivism and irrational paranoia. An important step forward is to improve awareness and education of all stakeholders about privacy, technical limitations to protect it, and building regulatory barriers to avoid discrimination.

## Conclusions

AI and big data in medicine are only in their childhood stages; they grow up fast. Whether they grow up well is still an open question that the future will answer. However, they will not grow up well without actively helping them do so. There are several important initiatives that contribute to this, such as the Global Alliance for Genomics and Health (GA4GH), an organization setting a policy and technical framework for respecting human rights to enable responsible genomic data sharing [74], or the European Union General Data Protection Regulation (GDPR) [75] that sets a completely novel privacy regulation for the European Union. Such initiatives are converging toward building a landscape that enables science while building trust in improving protection of individual rights. I invite the readers to visit the JMIR Open Access collections available on the Web on the following topics: “Big Data,” “Decision Support for Health Professionals,” and “Artificial Intelligence” [76-78].

## Abbreviations

AI: artificial intelligence
CPOE: computerized provider order entry
GA4GH: Global Alliance for Genomics and Health
GDPR: General Data Protection Regulation
RECIST: Response Evaluation Criteria In Solid Tumors
SNOMED CT: Systematized Nomenclature of Medicine - Clinical Terms
